# A Combination of Apple Vinegar Drink with *Bacillus coagulans* Ameliorates High Fat Diet-Induced Body Weight Gain, Insulin Resistance and Hepatic Steatosis

**DOI:** 10.3390/nu12092504

**Published:** 2020-08-19

**Authors:** Raquel Urtasun, Joana Díaz-Gómez, Miriam Araña, María José Pajares, María Oneca, Paloma Torre, Maddalen Jiménez, Germán Munilla, Miguel Barajas, Ignacio Encío

**Affiliations:** 1 Biochemistry Area, Department of Health Science, Public University of Navarre, 31008 Pamplona, Spain; raquel.urtasun@unavarra.es (R.U.); miriam.arana@unavarra.es (M.A.); mjose.pajares@unavarra.es (M.J.P.); maria.oneca@unavarra.es (M.O.); 2 Ecovinal S.L., Pol. Ind Gobella, 1, 31589 Sartaguda, Spain; jdiaz@ecovinal.com (J.D.-G.); gmunilla@ecovinal.es (G.M.); 3 Navarre’s Health Research Institute (IdiSNA), 31008 Pamplona, Spain; 4 Nutrition and Bromatology area, Department of Natural Sciences, Public University of Navarre, 31006 Pamplona, Spain; paloma@unavarra.es; 5 Division of Hematological-Oncology, CIMA, University of Navarre, 31006 Pamplona, Spain; mjimenezan@unav.es

**Keywords:** obesity, high fat diet, insulin resistance, hepatic steatosis, vinegar, *Bacillus coagulans*

## Abstract

Obesity is a worldwide epidemic characterized by excessive fat accumulation, associated with multiple comorbidities and complications. Emerging evidence points to gut microbiome as a driving force in the pathogenesis of obesity. Vinegar intake, a traditional remedy source of exogenous acetate, has been shown to improve glycemic control and to have anti-obesity effects. New functional foods may be developed by supplementing traditional food with probiotics. *B. coagulans* is a suitable choice because of its resistance to high temperatures. To analyze the possible synergic effect of Vinegar and *B. coagulans* against the metabolic alterations induced by a high fat diet (HFD), we fed twelve-week-old C57BL/6 mice with HFD for 5 weeks after 2 weeks of acclimation on a normal diet. Then, food intake, body weight, blood biochemical parameters, histology and liver inflammatory markers were analyzed. Although vinegar drink, either alone or supplemented with *B. coagulans*, reduced food intake, attenuated body weight gain and enhanced glucose tolerance, only the supplemented drink improved the lipid serum profile and prevented hepatic HFD-induced overexpression of CD36, IL-1β, IL-6, LXR and SREBP, thus reducing lipid deposition in the liver. The beneficial properties of the *B. coagulans*-supplemented vinegar appear to be mediated by a reduction in insulin and leptin circulating levels.

## 1. Introduction

The prevalence of obesity has increased worldwide in the last ~50 years, reaching epidemic proportions. Nowadays, it is one of the world’s greatest public health challenges because it comes associated with metabolic complications such as dyslipidemia and insulin resistance with a consequent risk of cardiovascular disease, non-alcoholic fatty liver disease (NAFLD) and other adverse health conditions [1]. Excess food intake, especially high-calorie diets, such as high-fat and high-sugar diets, is considered the greatest risk factor in the development of obesity [2]. In addition, over the last decade, different studies have been highlighting an increasingly more important role of the gut microbiota in the development of obesity. Many studies have reported differences in microbiota composition between obese and lean humans [3,4] and experimental studies have demonstrated that both short-and long-term dietary change can influence our microbiota by reducing bacterial diversity and modifying the microbial composition taxa [5]. However, potential mechanisms by which gut microbiota promote metabolic disorders and obesity are still not well understood. Normally, dietary polysaccharides that escape digestion in the small intestine are metabolized by the microbiota into short-chain fatty acids (SCFA) during fiber digestion. The most abundant SCFA are acetate, propionate and butyrate. Although some studies postulated that excess SCFA and bacterial genes related to polysaccharides metabolism presented in feces are associated with the obese phenotype [6], new evidence has also demonstrated that SCFA could also increase energy expenditure and anorexic hormone production and support a beneficial role for SCFA in carbohydrates and lipid metabolism, contributing to improved insulin sensitivity [7]. In addition, SCFA improve the gut health maintenance of intestinal barrier integrity, mucus production and protection against inflammation [7].

So far, obesity treatments such as exclusively calorie reduced intake and/or bariatric surgery modify the gut microbiota and are associated with health benefits. However, they have not been completed successfully in the long term. Both present restricted benefits, mostly because of complex and persevering hormonal response [8]. Consuming diets or foods with bioactive nutrients, such as fermented foods, has been proposed as an effective solution to improve the effectiveness of conventional treatments [9,10]. Some fermented foods are highly enriched in SCFA. Vinegar, widely used as a flavouring and preservative in aliments, is a fermented drink enriched in bioactive components such as acetic acid and polyphenolic compounds [11]. Besides, oral ingestion of vinegar increases circulation of acetate [12]. Multiple functional properties of the vinegar are well established, including antioxidant, anti-hyperglycaemia, anti-hypertension, anti-microbial, anti-thrombotic and even anti-cancer properties [13]. Moreover, in the last decades, vinegar has captured some interest for its beneficial effects on controlling body weight and visceral fat accumulation [11,14,15]. Mechanistically, acetate has been found to block fatty acid synthesis and reduce lipid content in liver and adipose tissue, improving obesity [16,17].

Potential sources of exogenous acetate include vinegar as well as the supplementation of acetogenic fiber and probiotics or lactic acid bacteria [18]. Among lactic acid bacteria, *Bifidobacterium* and *Lactobacillus* are the most studied and used probiotic microorganisms [19]. However, these microorganisms cannot survive heat treatment. *Bacillus* spores can survive severe processing conditions as well as tolerate and resist against the harsh conditions found in the gastrointestinal tract [20]. Although the use of probiotic strains of *Bacillus* is still recent, emerging studies have described the health benefits associated with their ingestion [20,21]. For instance, probiotic strains of *Bacillus* were shown to provide protective effects against high fat diet-induced metabolic disorders in mice [22] and anti-obesity effects [23]. However, relatively few studies have examined the possible synergistic effect between fermented foods and a specific *Bacillus* species. *B. coagulans* GBI-30 is a lactic producing, spore forming, bacteria with GRAS (generally recognized as safe) status [20]. Authorized for human usage, the strain, that transiently colonizes the intestine without the need for frequent consumption, has already been proven to have beneficial effects in a variety of disorders, including irritable bowel syndrome, colitis, rheumatoid arthritis, and common viral infections of the respiratory tract [24].

For this reason, the aim of this study was to investigate whether the combination of *B. coagulans* GBI-30 with a heat-processed organic drink that contains apple cider vinegar, will be useful to prevent the development of obesity and its metabolic alterations such as insulin resistance and fatty liver in a mouse model of diet-induced obesity.

## 2. Materials and Methods

### 2.1. Organic Vinegar Drink and Probiotic Strain

The organic vinegar drink was elaborated by Ecovinal S.L. (Sartaguda, Spain) and contains 5% organic raw apple cider, unfiltered vinegar and fruit juices. The vinegar dose used in our animal study was equivalent to the concentration of one tablespoon of vinegar diluted in 300 mL. This concentration was selected based on previous animal studies considered as low dose [25,26] and the recommended amount in human studies (1 tablespoon of vinegar per day) [27]. The complete nutritional analysis is shown in Table 1. After heat-treatment, the vinegar drinks were stored at room temperature until used. An organic drink of equal calorie content and formulation to apple vinegar drink without vinegar (Vehicle) was also produced by Ecovinal S.L. (Sartaguda, Spain) for the experimental animal procedure.

Freeze-dried spores of *B. coagulans* GBI-30 6086, kindly provided by Ganeden Inc. (Mayfield Heights, OH, USA) were added to vinegar drinks in order to achieve 10^9^–10^10^ spores per serving of the drink product (200 mL), i.e., approximately 10^7^–10^8^ spores/mL. This dose was selected based on two different rat studies that showed increased fecal SCFA [28] and no metabolic homeostasis affectation [20].

### 2.2. Animal and Experimental Design

Eight-week-old male C57BL/6J mice were purchased from Charles River Laboratories. Mice were maintained in a temperature (22 to 28 °C) and humidity-controlled room on a 12-h light/12-h dark cycle with free access to food and drink during all of the study. In order to study the effect of vinegar and *B. coagulans* in a diet-induced obesity mice model, mice at the age of 12 weeks and at an average weight of 26.0 g, were randomly assigned into 5 groups (*n* = 6 mice per group). Each group had free access to one of the following beverages:

(1) Water, (2) Organic drink without vinegar (Vehicle), (3) Vehicle with *B. coagulans* GBI-30 6086 (Vehicle_Bc), (4) Apple vinegar drink (Vinegar), (5) Apple vinegar drink supplemented with *B. coagulans* GBI-30 6086. (Vinegar_Bc). The different groups were acclimatized for 2 weeks with a normal standard pellet diet (ND).

**Figure 1:**
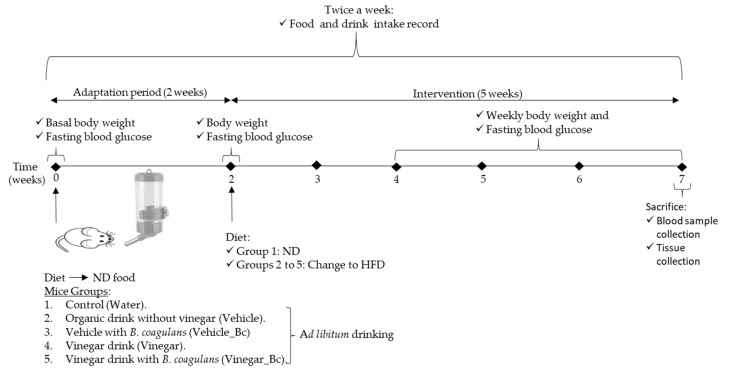
Animal groups and experimental design used in the study. HFD: High fat diet, ND: Normal rodent diet.

Then, group 1 remained on the ND while the other groups were fed with a High fat diet (HFD) (60% Kcal from fat Teklad Diet TD.06414, Envigo, Madison, WI, USA) for 5 additional weeks, as shown in the experimental design (Figure 1).

During the experimental study, food and drink intake were recorded 2 times per week and body weight was measured weekly. Drink was changed every 3 days to minimize contamination. At the end of the experimental period (7 weeks), animals were sacrificed by cervical dislocation after overnight fasting. All animal procedures were approved by the Institutional Committee on Care and Use of Laboratory Animals (CEEA, University of Navarra, Protocol number: 028-18).

### 2.3. Fasting Blood Glucose and Tolerance Test

Animals were fasted overnight, with free access to water prior to the test, and blood samples were obtained from the tip of the tail vein. Glucose concentrations were monitored using a pre-calibrated glucometer (Accu-chek Aviva, Roche, Basel, Switzerland). Fasting blood glucose (FBG) was recorded once a week.

In order to perform a glucose tolerance test (GTT), mice were fasted overnight, and 2 g/kg body weight of glucose was injected intraperitoneally. Measurement of blood glucose levels were performed at 0, 20, 40, 60, 90, and 120 min after glucose injection by collection of tail blood samples. The area under the curve (AUC) was assessed for each group from 0 to 120 min post glucose injection. The Homeostasis Model Assessment of Insulin Resistance (HOMA-IR), a widely used index for the assessment of insulin resistance [29], was calculated by the formula: HOMA-IR = serum C-peptide (nmol L^−1^) * serum glucose (mmol L^−1^)/22.5.

### 2.4. Serum Biochemical Analysis

Mice were fasted overnight before retro-orbital blood was drawn. Serum was obtained from blood samples after centrifugation for 15 min at 2200*× g* at 4 °C. Supernatant was collected and stored at −20 °C. Fasting serum cholesterol and triglycerides (TG) were determined using direct enzymatic colorimetric assays (Sigma-Aldrich, St. Louis, MO, USA). Total Free fatty acid (FFA) serum levels were quantified by means of a colorimetric assay using a commercial kit (Sigma-Aldrich, St. Louis, MO, USA).

### 2.5. C-Peptide, GLP-1 and Leptin Analysis

Serum C-peptide was assayed by sandwich ELISA method using an Ultra-Sensitive Mouse C-peptide ELISA kit (Abyntek Biopharma, Derio, Spain). GLP-1 levels in serum were determined using an ELISA kit (Sigma-Aldrich, St. Louis, MO, USA). Leptin levels were quantified using an enzyme-linked immunosorbent assay ELISA kit (Sigma-Aldrich, St. Louis, MO, USA).

### 2.6. Quantitative Real-Time PCR (RT-qPCR)

Total RNA was extracted from the livers of mice using a RNeasy Mini kit (Qiagen, Germantown, MD, USA). Then, cDNA synthesis (RT) was performed with RT Premix (Invitrogen, Carlsbad, CA, USA). Quantitative real-time PCR (qPCR) were achieved using IQ SYBR green (Bio-Rad, Hercules, CA, USA), in a CFX Connect ^TM^ Real-time system (Bio-Rad, Hercules, CA, USA) according to the manufacturer’s instructions. The primer pairs used are listed in Table 2. The mRNA levels were calculated as a ratio, using the 2^−∆∆*C*T^ method, for comparing the relative mRNA expression levels between different groups in the qPCR. All qPCRs were performed in triplicate and RPLPO gene was used to normalize gene expression.

### 2.7. Liver Histology and Morphometry

Tissue samples obtained from necropsy were fixed in 10% formalin for 24 h. The tissues were dehydrated, paraffin-embedded and processed for hematoxylin and eosin (H&E) staining. Digital images (ten per liver section) were acquired randomly with a 40× objective using a digital camera coupled to a conventional microscope (Olympus CH, Shinjuku, Japan).

The morphometric estimation of hepatic steatosis was analyzed with ImageJ software using digital images obtained and data expressed as a percentage of the area occupied by lipid droplets. For the quantification of the macrovesicular steatosis, two different observers, who were blinded for the treatment applied to each animal, individually examined the images and visually determined the percentage of hepatocytes containing macrovesicular lipid droplets. Macrovesicular steatosis was defined as lipid droplets larger than the cell nucleus.

### 2.8. Triglyceride Liver Content

First, liver tissue was homogenized in a 5% Nonidet P40 solution. After heating the samples to 100 °C in a water bath for 5 min, they were cooled to room temperature. Triglyceride content in the liver extracts was then determined using a Triglyceride Quantification Kit (Sigma-Aldrich, MO, USA). Obtained values were normalized with protein liver content.

### 2.9. Statistical Analysis

Data were expressed as the mean ± standard deviation (SD). Statistical analysis was performed using GraphPad Prism version 8 (GraphPad, La Jolla, CA, USA). The statistical analysis of body weight evolution and glucose tolerance test were performed by two-way repeated measures ANOVA followed by Bonferroni post hoc test. For all other data, one-way analysis of variance ANOVA or Kruskal–Wallis test followed by Tukey’s multiple comparison test was performed. The Student’s t or Mann–Whitney *U* test was used for the two groups’ comparisons. *p* values < 0.05 were considered as statistically significant.

## 3. Results

### 3.1. Vinegar Drink Either Alone or in Combination with B. coagulans Attenuated High Caloric Diet-Induced Weight Gain

**Figure 2:**
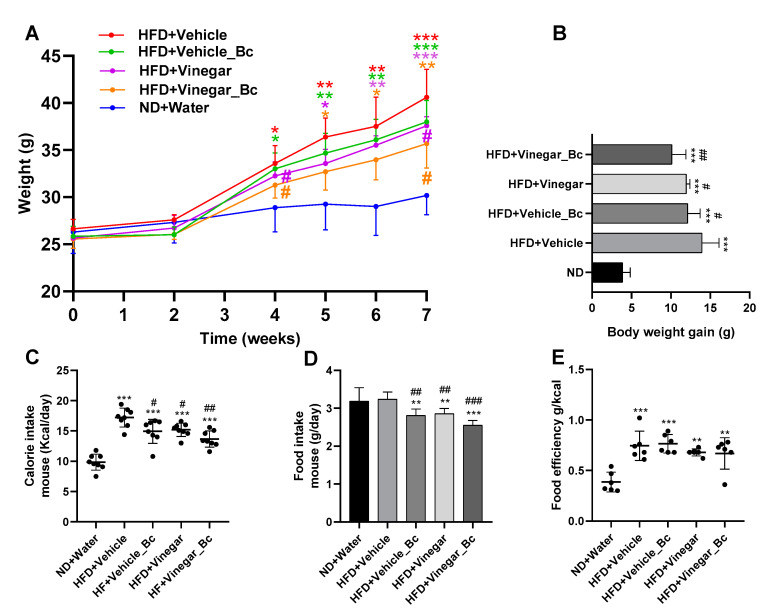
Effect of the different drinks on body weight, weight gain, food and calorie intake in HFD-induced obese mice. (**A**) Changes in body weight during the animal experimental study, (**B**) Differences in body weight gain between groups, (**C**) Daily energy intake calculated by converting the amount of food and drink consumed into calories, (**D**) Differences in food intake (g/mouse/day) between groups, (**E**) Metabolic or food efficiency ratio represented as the body weight gain relative to energy intake. Abbreviations. ND: Normal diet; HFD: High fat diet; ND+Water: Normal diet plus water; HFD+Vehicle: High fat diet plus vehicle (non-vinegar), HFD+Vehicle_Bc: High fat diet plus vehicle and *B. coagulans*; HFD+Vinegar: High fat diet plus vinegar; HFD+Vinegar_Bc: High fat diet plus vinegar and *B. coagulans.* Data are expressed as mean ± SD. * *p*  <  0.05, ** *p*  <  0.01, *** *p*  <  0.001 vs. ND group, ^#^ *p*  <  0.05, ^##^ *p*  <  0.01 ^###^ *p*  <  0.001 vs. HFD+Vehicle. (*n* = 6 mice per group).

In this study, we examined the effect of an Apple vinegar drink, either alone or in combination with *B. coagulans*, in mice fed with a HFD. For this purpose, male C57BL/6 mice were divided into five groups (six mice per group) with free access to the above described drinks of equal calorie content, except for the ND group (water control group) (Figure 1). During the entire HFD feeding period, mice body weight was significantly higher in every HFD group than in the ND group (Figure 2A). Though increased, total body weight at the end of the experimental procedure showed lower increments in the HFD+Vinegar and HFD+Vinegar_Bc groups than in the non-vinegar obese mice group (HFD+Vehicle) (*p* < 0.05). This significant attenuation of total body weight was already observed from week 5 of the experimental design (Figure 2A). In accordance with this result, an increase in body weight gain in mice fed with HFD (*p* < 0.001) was observed at the end of the experimental period (Figure 2B), and a significant reduction in this increase was observed in the HFD+Vehicle_Bc, HFD+Vinegar, HFD+Vinegar_Bc groups when compared to their corresponding control obese mice (HFD+Vehicle) (*p* < 0.05 for all except *p* < 0.01 for HD+Vinegar_Bc) (Figure 2B).

Mean energy intake was estimated by converting the amount of food and drink consumed into calories using the information provided by the Tekland diet manufacturer (Envigo, Madison, WI, USA) and the calorie content of the vinegar drink (Table 1). As shown in Figure 2C, daily energy intake was higher in HFD mice than in the ND mice (*p* < 0.001). However, this increase was significantly smaller in HFD+Vehicle_Bc, HFD+Vinegar and HFD+Vinegar_Bc groups than in the HFD+Vehicle group (*p* < 0.05, *p* < 0.05, *p* < 0.01, respectively). This result may be attributable to a reduction in mice food intake, as the daily food consumption (grams) in HFD+Vehicle_Bc, HFD+Vinegar and HFD+Vinegar_Bc was lower than in the HFD+Vehicle and ND groups (*p* < 0.05, *p* < 0.05, *p* < 0.001, respectively) (Figure 2D). However, there were no significant differences between HFD groups in drink intake (not shown), nor in the metabolic efficiency ratio (body weight gain relative to mean energy intake) among the HFD groups (Figure 2E).

### 3.2. Vinegar Drink Either Alone or in Combination with B. coagulans Improves Glycemic and Glucose Tolerance

**Figure 3:**
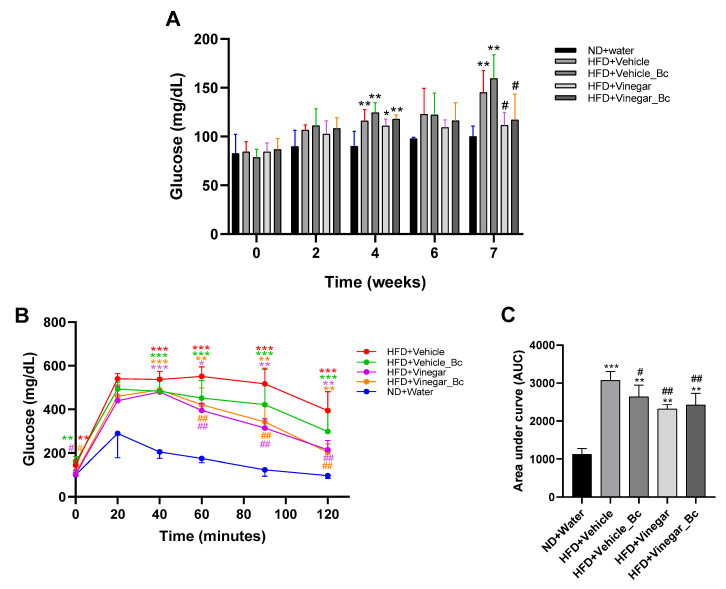
Vinegar and *B. coagulans* improved glucose homeostasis. (**A**) Fasting blood glucose measured at several time points during the experimental procedure, (**B**) Blood glucose levels in response to glucose tolerance test at week 7, (**C**) Effect on area under the curve (AUC). Abbreviations. ND: Normal diet; HFD: High fat diet; ND+Water: Normal diet plus water; HFD+Vehicle: High fat diet plus vehicle (non-vinegar), HFD+Vehicle_Bc: High fat diet plus vehicle and *B. coagulans*; HFD+Vinegar: High fat diet plus vinegar; HFD+Vinegar_Bc: High fat diet plus vinegar and *B. coagulans.* Data are expressed as mean ± SD. * *p* < 0.05, ** *p* < 0.01, *** *p* < 0.001 vs. ND, ^#^ *p* < 0.05, ^##^ *p* < 0.01 vs. HFD+Vehicle group. (*n* = 6 mice per group).

To explore the effect of vinegar ± *B. coagulans* on glucose homeostasis, basal serum glucose was measured at several time points during the experimental procedure, and a GTT was performed at the end of the experiment. No significant changes were detected in serum glucose level among the groups before week 4 of the experimental study. However, at week 4 all HFD groups displayed higher basal glucose levels than the ND group (*p* < 0.001 for HFD+Vehicle, HFD+Vehicle_Bc and HFD+Vinegar_Bc groups, *p* < 0.05 for HFD+Vinegar) (Figure 3A). Interestingly, after seven weeks only the HFD+Vehicle and HFD+Vehicle_Bc exhibited higher fasting glucose levels than the ND group (*p* < 0.01). Moreover, significantly smaller fasting blood glucose levels were found in the HFD+Vinegar and HFD+Vinegar_Bc mice than in the HFD+Vehicle group (*p* < 0.05) (Figure 3A).

In regard to the GTT performed at week 7, as expected, basal serum glucose concentration was higher in the HFD+Vehicle and HFD+Vehicle_Bc mice than in the ND group (*p* < 0.01). In contrast, the basal glucose concentration in HFD+Vinegar and HFD+Vinegar_Bc group was lower than in the HFD+Vehicle group (*p* < 0.05) (Figure 3B). Peak levels of glucose were reached in every group 20 min after the intraperitoneal administration of glucose. Glucose levels remained higher in the HFD+Vehicle mice than in the ND mice 40, 60, 90 and 120 min after injection (*p* < 0.001) (Figure 3B).

In contrast, glucose levels in the HFD+Vinegar and HFD+Vinegar_Bc groups decreased faster from minute 60 to 120, normalizing their values compared to HFD+Vehicle (*p* < 0.01). A trend towards normalization was also observed in the HFD+Vehicle_Bc mice, though in this case the decrease did not reach statistical significance (Figure 3B). Besides, the area under the curve (AUC) was greater in all HFD mice than in the ND mice (*p* < 0.001 for HFD+Vehicle, and *p* < 0.01 for the rest of HFD groups) (Figure 3C), indicating impaired glucose tolerance. However, HFD+Vehicle_Bc, HFD+Vinegar and HFD+Vinegar_Bc presented smaller AUC than the HFD+Vehicle mice (*p* < 0.05, *p* < 0.01, *p* < 0.01, respectively), pointing to a better glucose tolerance for mice in these groups than for those in the HFD control group (Figure 3C).

### 3.3. Vinegar Drink Together with B. coagulans Attenuates Dislipidemia in Obese Mice

**Figure 4:**
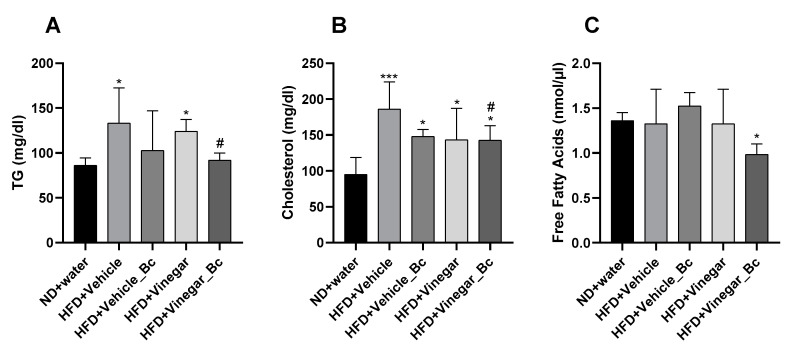
Serum lipid profile in HFD-fed mice supplemented with vinegar and non-vinegar drinks ± *B. coagulans* for 5 weeks. (**A**) Concentration of serum Triglycerides (TG), (**B**) Serum total cholesterol, (**C**) and serum free fatty acids (FFA) on mice fed ND or HFD, respectively. Abbreviations. ND: Normal diet; HFD: High fat diet; ND+Water: Normal diet plus water; HFD+Vehicle: High fat diet plus vehicle (non-vinegar), HFD+Vehicle_Bc: High fat diet plus vehicle and *B. coagulans*; HFD+Vinegar: High fat diet plus vinegar; HFD+Vinegar_Bc: High fat diet plus vinegar and *B. coagulans.* Values are expressed as the mean ± SD. * *p* < 0.05, *** *p* < 0.001 vs. ND, ^#^ *p* < 0.05 vs. HFD+Vehicle group. (*n* = 6 mice per group).

To assess the effect of vinegar ± *B. coagulans* on lipid profile in mice fed HFD, triglycerides, cholesterol and free fatty acids (FFA) levels were determined in serum. After 5 weeks of HFD feeding, a significant increase in serum triglyceride levels was quantified in HFD+Vehicle and HFD+Vinegar groups compared to the ND group (*p* < 0.05), thus suggesting the onset of dyslipidemia by HFD feeding (Figure 4A). As shown in the figure, supplementation of HFD with Vinegar and *B. coagulans* prevented this increase (HFD+Vinegar_Bc vs. HFD+Vehicle: *p* < 0.05).

In this dyslipidemic condition, cholesterol levels were significantly higher in all HFD groups (HFD+Vehicle, HFD+Vehicle_Bc, HFD+Vinegar, HFD+Vinegar_Bc) than in the ND group (*p* < 0.001 for HFD+Vehicle and *p* < 0.05 for the rest of the HFD groups). Only the HFD+Vinegar_Bc group significantly ameliorated cholesterol levels compared to the HFD+Vehicle group (*p* < 0.05) (Figure 4B). In addition, there were no significant differences in fasting serum FFA between the HFD+Vehicle, HFD+Vehicle_Bc, HFD+Vinegar and ND groups. However, a significant reduction was found in FFA serum concentration of the HFD+Vinegar_Bc when comparing it to the control group (*p* < 0.05) (Figure 4C).

### 3.4. Vinegar Supplemented with B. coagulans Ameliorated Fat Liver Accumulation: Histological Assessment of the Liver

**Figure 5:**
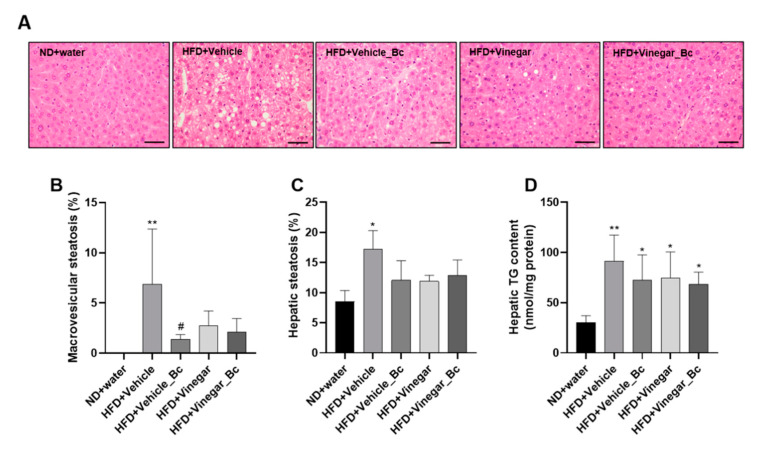
Effect of vinegar and *B. coagulans* on hepatic histology and TG liver accumulation in HFD-induced obese mice. (**A**) Representative images of H&E-stained liver sections from different animal experimental groups are shown (40 × magnification), (**B**) Percentage of macrovesicular steatosis, (**C**) Percentage of hepatic steatosis (**D**) and hepatic TG content on mice fed ND or HFD, respectively. Scale bar: 50 µm. Abbreviations. ND: Normal diet; HFD: High fat diet; ND+Water: Normal diet plus water; HFD+Vehicle: High fat diet plus vehicle (non-vinegar), HFD+Vehicle_Bc: High fat diet plus vehicle and *B. coagulans*; HFD+Vinegar: High fat diet plus vinegar; HFD+Vinegar_Bc: High fat diet plus vinegar and *B. coagulans.* Values are expressed as the mean ± SD. * *p* < 0.05, ** *p* < 0.01 vs. ND, ^#^ *p* < 0.05 vs. HFD+Vehicle group. (*n* = 6 mice per group).

Photomicrographs of liver tissue for all animal experimental groups are shown in (Figure 5A). At the end of the animal study, the liver tissue of mice fed with ND presented a normal architecture of hepatocytes, with no appearance of lipid droplet deposition (Figure 5A). In contrast, HFD-fed mice showed fat droplet deposition consisting of mixed microvesicular and macrovesicular steatosis, while vinegar and *B. coagulans*, alone or in combination, remarkably suppressed vacuole formation and lipid accumulation in hepatocytes (Figure 5A). The histopathological quantification showed a higher degree of macrovesicular steatosis in HFD+Vehicle mice than in the ND group (*p* < 0.01) (Figure 5B). Interestingly, vinegar drink, *B. coagulans* and the combination of both exhibited less macrovesicular deposition, though a statistically significant change was observed only in the HFD+Vehicle_Bc group (*p* < 0.05) (Figure 5B). The quantification of liver micro and macrovesicular steatosis yielded a higher number of vesicles in the HFD+Vehicle group than in the ND (*p* < 0.05) (Figure 5C). For the rest of the HFD groups, no significant differences in micro and macrovesicular steatosis with the ND group were found (Figure 5C). Consistent with this histological and morphological observation, hepatic TG accumulation appeared to be higher in mice fed with HFD compared to ND (*p* < 0.01 for HFD+Vehicle, and *p* < 0.05 for the rest of the HFD groups) (Figure 5D). Supplementation of HFD with vinegar, *B. coagulans*, or their combination did not significantly counteract hepatic TG accumulation, although a trend towards a decrease was observed in every case. In summary, our results showed that vinegar, and *B. coagulans* alone or in combination, may reduce fat liver accumulation induced by the HFD and improve liver steatosis.

### 3.5. Vinegar Drink Together with B. coagulans Ameliorates the Expression of Altered Lipid Metabolism-Related and Pro-Inflammatory Genes in the Livers of High Fat Diet Mice

**Figure 6:**
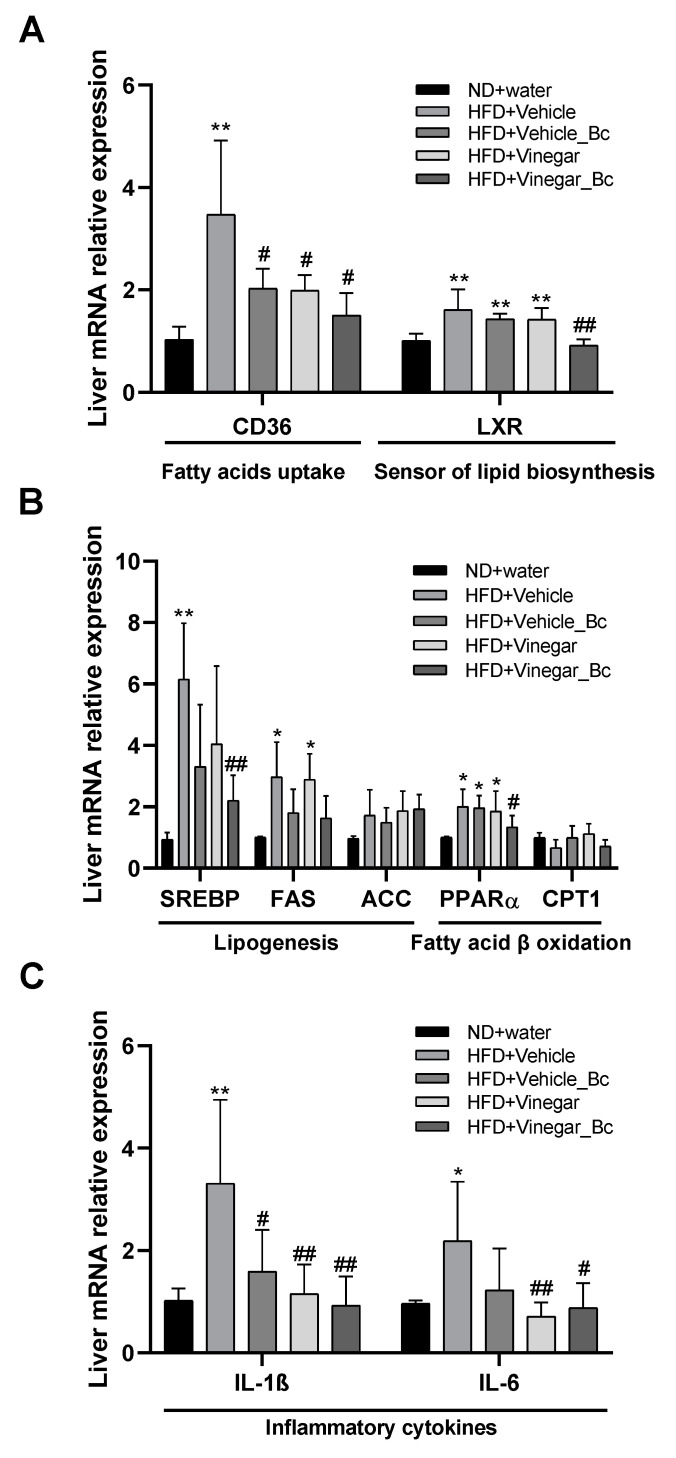
Effect of vinegar ± *B. coagulans* on mRNA levels of hepatic genes. (**A**,**B**) Effect of vinegar ± *B. coagulans* on mRNA levels of several genes involved in hepatic lipogenesis and β-oxidation, (**C**) Effect of vinegar ± *B. coagulans* on mRNA expression levels of pro-inflammatory cytokines in the liver. Total RNA extracted from the liver was reverse transcribed, and each gene expression was quantified by real-time PCR using gen-specific primers. All genes were normalized to expression of RPLPO. The relative expression level of each gene was calculated as the fold change from the triplicates in each group using the 2^−ΔΔ*C*t^ method. The y-axis represents the relative expression of the studied genes. Gene full names. RPLPO: Ribosomal protein P0; LXR: Liver X receptor; CD36: Cluster of differentiation 36; PPARα: Peroxisome proliferator activated receptor; SREBP: sterol regulatory element-binding protein; FASN: Fatty acid synthase; IL-1β: Interleukin 1 beta; IL-6: Interleukin 6; CPT-1: Carnitine palmitoyltransferase; ACC: Acetyl-CoA-carboxylase. Abbreviations. ND: Normal diet; HFD: High fat diet; ND+Water: Normal diet plus water; HFD+Vehicle: High fat diet plus vehicle (non-vinegar), HFD+Vehicle_Bc: High fat diet plus vehicle and *B. coagulans*; HFD+Vinegar: High fat diet plus vinegar; HFD+Vinegar_Bc: High fat diet plus vinegar and *B. coagulans.* Data are expressed as mean ± SD. * *p* < 0.05, ** *p* < 0.01 vs. control, ^#^ *p* < 0.05, ^##^ *p* < 0.01 vs. HFD+Vehicle group. (*n* = 6 mice per group).

Fatty liver occurs when fat accumulation is caused by impaired fatty acid oxidation along with increased lipid synthesis. To understand the molecular mechanisms underlying the protective effect observed in the liver for the vinegar drink supplemented with *B*. *coagulans*, we next evaluated the expression in liver tissue of lipid metabolism-related genes, such as lipogenic genes, fatty acid synthesis genes or fatty acid oxidation genes (Figure 6). As shown in the Figure, at the end of the experimental study we found a significant increase in the expression of the adipogenesis-related gene CD36 in the liver of mice fed with HFD+Vehicle compared to mice fed with ND (*p* < 0.01). Figure 6A also shows that supplementation of HFD with either vinegar, *B. coagulans* or both prevented HFD from inducing long chain fatty acid receptor CD36 expression (*p* < 0.05 compared to HFD+Vehicle), thereby attenuating fatty acid uptake in the liver. Similar results were found when looking for LXR sensor of cholesterol metabolism and lipid biosynthesis expression: LXR mRNA expression was higher in the HFD+Vehicle, HFD+Vehicle_Bc and HFD+Vinegar groups than in the ND group (*p* < 0.01). However, supplementation of HFD with Vinegar + *B. coagulans* prevented induction of LXR by HFD (HFD+Vinegar_Bc vs. HFD+Vehicle: *p* < 0.01) and maintained normal LXR expression levels.

Plasma fatty acid taken up by the liver is further augmented by increased hepatic de novo lipogenesis. LXR directly regulates the expression of genes involved in fatty acid synthesis including SREBP, a master regulator of fatty acid synthesis. Thus, not surprisingly, HFD feeding induced a significant upregulation of SREBP in mice fed with HFD+Vehicle (HFD+Vehicle vs. ND: *p* < 0.01) (Figure 6B). Upregulation was attenuated by both vinegar and *B. coagulans*, though only reached statistical significance when the two were present (HFD+Vinegar_Bc vs. HFD+Vehicle: *p* < 0.01) (Figure 6B).

Regulation of de novo lipogenesis occurs too by the regulation of various key enzymatic activities, including FAS and ACC among others. Analysis of the hepatic expression of these genes revealed no statistically significant differences in ACC expression among groups (Figure 6B). However, HFD feeding increased FAS expression (HFD+Vehicle vs. ND: *p* < 0.05). In this case, neither vinegar nor *B. coagulans* supplementation led to a significant attenuation of HFD-induced FAS expression (Figure 6B).

Several studies have shown that PPARα expression is activated by fatty acids. This is especially important during fasting, when plasma FFAs and the flux of fatty acids through the liver increase dramatically [30]. Accordingly, in our experimental study HFD feeding induced a significant upregulation of PPARα compared to ND (*p* < 0.05), and in consonance with the reduction in FFA serum concentration described above, HFD-induced PPARα upregulation was prevented by HFD+Vinegar_Bc (HFD+Vinegar_Bc vs. HFD+Vehicle: *p* < 0.05) (Figure 6B). Besides, HFD feeding impaired the fatty acid oxidation pathway as pointed out by the slight decrease observed in mRNA abundance of CPT1 in HFD-fed mice, which was restored in HFD+Vehicle_Bc and HFD+Vinegar, although this last result did not reach statistical significance (Figure 6B).

Obesity is closely associated with an increased risk of NAFLD that includes a disease spectrum that ranges from steatosis or isolated fatty liver to non-alcoholic steatohepatitis (NASH), a more aggressive form that can progress to fibrosis, cirrhosis and hepatocarcinoma [31]. Numerous studies have reported complex interactions between obesity, hepatic steatosis and chronic inflammation. In addition, adipose tissue dysfunction and the hepatic inflammatory response have an important role in the transition from isolated fatty liver or steatosis to NASH. To examine whether vinegar drink ± *B. coagulans* improved liver inflammation in HFD-fed mice, the amount of mRNA of pro-inflammatory cytokines in the liver was determined. As shown in Figure 6C, HFD feeding induced a significant up-regulation of IL-1β and IL-6 mRNA (HFD+Vehicle vs. ND: *p* < 0.001 and *p* < 0.05, respectively). Supplementation of HFD with either vinegar, *B. coagulans* or both prevented Il-1β upregulation (HFD+Vehicle_Bc vs. HFD+Vehicle, *p* < 0.05, HFD+Vinegar vs. HFD+Vehicle: *p* < 0.01, and HFD+Vinegar_Bc vs. HFD+Vehicle: *p* < 0.01) in the liver (Figure 6C). Similarly, liver mRNA expression of IL-6 was significantly reduced in HFD+Vinegar and HFD+Vinegar_Bc mice, compared with HFD+Vehicle mice (*p* < 0.001 and *p* < 0.05, respectively) (Figure 6C).

### 3.6. Effect of Vinegar Drink and B. coagulans on Leptin, GLP-1 and Insulin Levels in Serum

Emerging evidence has shown an important role of the microbiota–gut–brain axis in the control of food intake by the hypothalamus and in insulin and leptin signaling in the contribution to evolution of obesity [32]. As mentioned before, mice fed with a HFD supplemented with vinegar ± *B. coagulans* gained less weight and had reduced food intake than non-vinegar HFD-fed mice. For this reason, we explored whether vinegar ± *B. coagulans* improved the response to anorexigenic hormones such as leptin, insulin and glucagon-like peptide 1 (GLP-1) in serum of HFD-fed mice.

**Figure 7:**
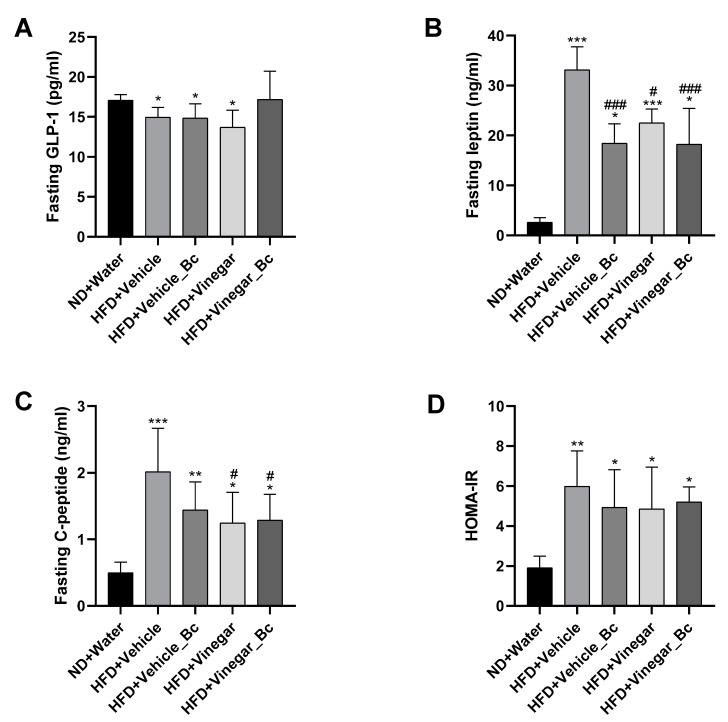
Effect of vinegar ± *B. coagulans* on GLP-1, Leptin and C-peptide in serum. (**A**) Fasting serum GLP-1, (**B**) Fasting serum leptin, (**C**) Fasting C-peptide levels, (**D**) Insulin sensibility estimated using average HOMA-IR in each group calculated from fasting serum glucose and fasting serum C-peptide concentrations with the formula: serum C-peptide (nmol L^−1^) * serum glucose (mmol L^−1^)/22.5. Abbreviations. ND: Normal diet; HFD: High fat diet; ND+Water: Normal diet plus water; HFD+Vehicle: High fat diet plus vehicle (non-vinegar), HFD+Vehicle_Bc: High fat diet plus vehicle and *B. coagulans*; HFD+Vinegar: High fat diet plus vinegar; HFD+Vinegar_Bc: High fat diet plus vinegar and *B. coagulans* Data are expressed as mean ± SD. * *p*  <  0.05, ** *p*  <  0.01, *** *p*  <  0.001 vs. ND group, ^#^ *p*  <  0.05, ^###^ *p*  <  0.001 vs. HFD+Vehicle. (*n* = 6 mice per group).

Apart from the well-known effect of fasting GLP-1 as an anorexic hormone able to reduce appetite and food intake [33], growing evidence has suggested that the reduction in plasma GLP-1 levels is related to disorders of glucose homeostasis [34]. In addition, recent studies have demonstrated that fasting circulating SCFA (acetate) are related to insulin sensitivity, lipolysis and fasting GLP-1 concentration in human blood [35]. To understand whether the mechanism underlying the effect of vinegar and *B. coagulans* to inhibit food intake and improve glucose homeostasis, could be related to the production of this anorexic hormone, fasting serum GLP-1 levels were explored. Figure 7A shows that mice receiving HFD had reduced serum fasting GLP-1 concentration than ND mice (*p* < 0.05). Neither vinegar nor *B. coagulans* supplementation counterparted this effect. However, no significant differences in fasting serum GLP-1 level were detected between the HFD+Vinegar_Bc and ND mice (Figure 7A).

As shown in Figure 7B, HFD mice exhibited significantly higher levels of leptin in serum than ND mice (*p* < 0.001 for HFD+Vehicle and HFD+Vinegar, *p* < 0.05 for the rest of the HFD groups), indicating leptin resistance in HFD-fed mice. Supplementation of HFD with either vinegar, *B. coagulans* or both decreased leptin serum levels (HFD+Vehicle_Bc vs. HFD+Vehicle: *p* < 0.001, HFD+Vinegar vs. HFD+Vehicle: *p* < 0.05, and HFD+Vinegar_Bc vs. HFD+Vehicle: *p* < 0.001), suggesting that the administration of vinegar ± *B. coagulans* to HFD mice improved leptin sensibility as evidenced by reduced food intake.

As shown before, the mean values of AUC obtained during GTT were greater in HFD-fed mice, thus indicating insulin resistance. In accordance with this, fasting serum C-peptide levels were elevated in HFD mice compared to ND mice (*p* < 0.001 for HFD+Vehicle, *p* < 0.01 for HFD+Vehicle_Bc and *p* < 0.05 for HFD+Vinegar and HFD+Vinegar_Bc) (Figure 7C). Supplementation of HFD with vinegar attenuated elevation (HFD+Vinegar and HFD+Vinegar_Bc groups vs HFD+Vehicle: *p* < 0.05). These results partially correlated with the insulin resistance index based in C-peptide (HOMA-IR) that showed a significant increase in all HFD-fed groups when compared to the ND mice (*p* < 0.01 for HFD+Vehicle and *p* < 0.05 for the rest of HFD groups). However, the resistance index was not restored by vinegar, *B. coagulans*, or their combination, although a trend towards a decrease was observed in every case (Figure 7D).

## 4. Discussion

Obesity is a prevalent health issue worldwide [36,37]. Associated with modernization, it affects physical activity and food habits. Consuming high fat diets has been identified as the main important reason for the increased risk of developing obesity [2]. Initially, apart from genetics and environmental factors, obesity was only attributed to a prolonged imbalance between energy intake and energy expenditure [38]. Nowadays, numerous studies in mice and humans have also shown that obesity is associated with changes in the diversity and abundance of the microbiota [5,6]. In addition, growing evidence has demonstrated that the microbiota may impact body weight gain and adiposity through a network of interconnected pathways, such as energy harvest and production of microbial metabolites, inflammatory response, and effects on the gut–brain axis. SCFA metabolites participate in this complex signaling [7].

Fermented foods, especially vinegars produced for different fruits, have long being consumed worldwide to obtain different health benefits, including body weight control [11]. Vinegar is considered a dietary source of acetate, due to its ingestion acetate levels in plasma increasing rapidly [39]. Nowadays, new functional foods are increasing their popularity due to their wide-range and long-term benefits [40]. Specially, people are interested in weight loss shifting away from “magic pills” and traditional weight loss programs to more natural and healthier alternatives. In line with this idea, the purpose of this study was to explore the role of a new functional drink, obtained from an organic apple vinegar beverage inoculated with the spore-forming *B. coagulans* as a probiotic in diet-induced obese mice.

*B. coagulans* is an ideal choice to develop functional foods because of its resistance to high temperatures. Although, there are still few studies showing the effect of *B. coagulans* on microbiota, several beneficial effects have been reported. Recently, it has been published that the administration of *B. coagulans* to rats resulted in a drastic increase in acetate and butyrate in rat feces, with a relatively higher level of total SCFA compared to untreated control rats [28]. In the present study, we found that both vinegar drink alone and vinegar drink supplemented with *B. coagulans* improved glucose tolerance and prevented body weight gain in a mice model of HFD-induced obesity. It is worth noting that we also found a protective effect on lipid profile, decreased expression of genes involved in lipogenesis, and a reduction in macrovesicular hepatic steatosis only when the organic drink was supplemented with both vinegar and *B. coagulans*. The ability of *B. coagulans* to increase intestinal acetate and butyrate concentrations [28], thus improving the effect of vinegar, is a likely explanation for this finding.

In the search for the mechanism involved in the control of weight gain, the impact of vinegar and *B. coagulans* on plasma leptin, insulin and Glucagon-like peptide 1 (GLP-1) was explored. GLP-1 is known for its glucoregulatory and appetite-suppressing effects, playing an important role in the regulation of food intake and insulin secretion [41]. Several studies have demonstrated that high fat diets impair the secretory function of GLP-1-producing cells [42,43]. More concretely, a reduction in GLP-1 expression was observed in diet-induced obese mice characterized by mitochondrial stress in colon enterocytes [44]. By contrast, in enteroendocrine L-cells, SCFA increase GLP-1 expression and secretion by activation of GRP43, mainly by the effect of dietary fibers [45]. According to these observations, we detected a reduction in fasting serum GLP-1 in HFD-induced obese mice. Interestingly, fasting serum GLP-1 levels were not reduced in HFD+Vinegar_Bc-induced obese mice, which suggests that GLP-1 regulation of appetite is involved in the effect of vinegar and *B. coagulans* on food intake and body weight. Thus, *B. coagulans*-derived SCFA, likely acetate, may contribute to the vinegar activity in the regulation of GLP-1 in vivo, although this needs further investigation.

Leptin is mainly produced by adipocytes, and serum HFD-induced levels of leptin correlate with adipocyte fat storage [46]. Acting in hypothalamic centers, major roles of leptin are the regulation of food intake and increasing energy expenditure. Leptin resistance occurs in response to high fat diets. In obesity, circulating levels of leptin rise, although its ability to reduce food intake and to increase energy expenditure diminishes; hyperleptinaemia and inflammation have been postulated as causative mechanisms [47]. Using different mouse models, Koch et al. demonstrated: first, that 6-weeks treatment of obese wild type mice with a leptin neutralizing antibody drives a 10% reduction in food intake; second, that hyperleptinaemia is a driving force of metabolic disorders; third, that a partial restoration of leptin levels in plasma leads to improved leptin sensibility and insulin sensitivity, and reduces weight gain and hepatic steatosis [48]. Recently, the same authors reported that preserving lower leptin levels in an obesogenic environment is highly beneficial for obesity and diabetes control. In this last study, partial leptin deficiency led to a better leptin sensibility in HFD mice, as assessed by an overall reduced food intake [49]. In concordance with these studies, in this work we found that a vinegar drink, either alone or supplemented with *B. coagulans*, partially reduced circulating leptin levels, improved glucose tolerance, decreased C-peptide levels in serum, and improved the insulin sensitivity index in HFD-induced obese mice. Normally, lower body weight and better glucose tolerance correlate positively, both in animal models and in human clinical studies. For this reason, we speculate that the observed beneficial effects obtained with vinegar and *B. coagulans*, such as improved glucose tolerance and even reduced fatty liver, could be in part explained by its ability to restore leptin sensibility, thus reducing food intake and body weight gain.

Fatty liver disease is associated with insulin resistance and obesity and may be considered as the hepatic manifestation of metabolic syndrome. While the physiopathology of the non-alcoholic fatty liver disease (NAFLD) is not fully understood, steatosis is considered as a key initial event. Lipotoxicity, insulin resistance and inflammation are essential players in the progression towards a non-alcoholic liver steatohepatitis (NASH), the more aggressive form of NAFLD [50]. Obesity-associated chronic inflammation is known to be responsible for the impaired insulin response. However, the role of pro-inflammatory cytokines in NAFLD is still unclear, although several studies have demonstrated that TNFα, IL-1β and IL-6 are important mediators in the development of NAFLD. In addition to their expected role as pro-inflammatory cytokines in inflammation, they promote steatosis, inflammation and fibrosis. For instance, IL-1β can stimulate the production of TNFα and IL-6, and increases the accumulation of triglycerides and cholesterol [51]. In the present work, we found that vinegar supplemented with *B. coagulans* improved the lipid profile and suppressed hepatic steatosis induced by HFD in mice. This protective effect seems to be mediated by a reduction in the hepatic expression of CD36, a protein involved in fatty acid uptake. Disruption of hepatic CD36 has been shown to reduce both lipid content and free fatty acid uptake in the liver of HFD-fed mice, and also to protect against associated systemic inflammation and insulin resistance [52]. According to these results, we also found that vinegar, *B. coagulans* and their combination suppressed the HFD-induced overexpression of IL-1β and IL-6 inflammatory cytokines in the liver. They also reduced HFD-induced overexpression of SREBP and LXR transcription factors. LXR is a nuclear receptor that controls lipid metabolism by regulating the expression of SREBP and other genes involved in hepatic lipogenesis in NAFLD [53]. In the liver, SREBP is involved in the biogenesis of cholesterol, fatty acids and triglycerides; it also has a role in the pathogenesis of NAFDL and NASH [54]. Activated by downstream signaling, SREBP increases FAS mRNA expression, which collaborates in fatty acids synthesis. Down-regulation of SREBP expression is thus a likely explanation for the reduction in both fat accumulation in the liver and cholesterol and triglycerides levels in serum observed in the HFD+Vinegar_Bc mice. Additionally, activation of LXR has also been described to have anti-inflammatory effects in steatosis [55,56]. Therefore, the reduction in LXR expression could reflect the effect of vinegar and *B. coagulans* on systemic inflammation. All together, these results are consistent with the alleviated liver hepatic fat accumulation induced by vinegar and *B. coagulans* in our mice model of diet-induced obesity

In the context of NAFLD, hepatic steatosis can be stimulated via increased fatty acid uptake, increased de novo lipogenesis and failed fatty acid oxidation. In the present work, we did not find a clear modulation of the hepatic expression of the lipogenic genes FAS and ACC by vinegar and *B. coagulans.* In contrast, our data show that vinegar and *B. coagulans,* independently, tended to enhance hepatic oxidation since they prevent the HFD-induced down-regulation of CPT1. Surprisingly, the expression of PPARα, a gene known to promote fatty acid oxidation, was increased in every HFD-fed group except for the HFD+Vinegar_Bc mice. Since hepatic fasting has been shown to induce PPARα expression [57] and considering that we collected liver hepatic tissue after an eight-hour fast, our animal experimental procedure could be the reason for this result. For this reason, further experiments are needed to better characterize the action of vinegar and *B. coagulans* on the beta-oxidation pathway in the liver.

## 5. Conclusions

In summary, our study demonstrates that a new functional organic drink containing apple cider vinegar reduced food intake, attenuated body weight gain and enhanced glucose tolerance. Moreover, when supplemented with *B. coagulans* at a concentration of approximately 10^7^–10^8^ spores/mL, the drink also improved the lipid serum profile and prevented hepatic steatosis induced by HFD in mice. This protective effect was mediated by a reduction in the hepatic expression of CD36, IL-1β, IL-6, LXR and SREBP, which alleviated fat accumulation in the liver. The ability of the vinegar drink to partly restore leptin and insulin sensibilities could explain its beneficial effects. Further experiments are needed to confirm whether *B. coagulans*-derived SCFA contribute or not to the vinegar activity.

## Figures and Tables

**Table 1:** Nutritional analysis of organic apple vinegar drink.

Component	Unit	Amount
Energy value	Kcal/100 mL	24
Energy value	KJ/100 mL	100
Fat	g/100 mL	<0.05
Saturated Fat	g/100 mL	<0.05
Monounsaturated fat	g/100 mL	<0.05
Polyunsaturated fat	g/100 mL	<0.05
Trans fat	g/100 mL	<0.10
Total sugar	g/100 mL	5
Glucose	g/100 mL	2.1
Fructose	g/100 mL	2.9
Sucrose	g/100 mL	<1
Maltose	g/100 mL	<1
Lactose	g/100 mL	<1
Fiber	g/100 mL	<0.05
Protein	g/100 mL	0.11
Sodium	mg/100 mL	8.7
Ash	g/100 mL	0.18
Moisture	g/100 mL	93.8

**Table 2:** Primer sequence for qPCR.

Gene		Sequence
RPLPO	Forward	AACATCTCCCCCTTCTCCTT
	Reverse	GAAGGCCTTGACCTTTTCAG
LXR	Forward	CTCAATGCCTGATGTTTCTCCT
	Reverse	TCCAACCCTATCCCTAAAGCAA
CD36	Forward	CACAGCTGCCTTCTGAAATGTGTGG
	Reverse	TTTCTACGTGGCCCGGTTCTAATTC
PPARα	Forward	ACTGGTAGTCTGCAAAACCAAA
	Reverse	AGAGCCCCATCTGTCCTCTC
SREBP	Forward	CACTTCATCAAGGCAGACTC
	Reverse	CGGTAGCGCTTCTCAATGGC
FASN	Forward	AGCCATGGAGGAGGTGGTGAT
	Reverse	GTGTGCCTGCTTGGGGTGGAC
IL-1β	Forward	TCGCTCAGGGTCACAAGAAA
	Reverse	CATCAGAGGCAAGGAGGAAAAC
IL-6	Forward	ACAAGTCGGAGGCTTAATTACACAT
	Reverse	TTGCCATTGCACAACTCTTTTC
CPT-1	Forward	TCTAGGCAATGCCGTTCAC
	Reverse	GAGCACATGGGCACCATAC
ACC	Forward	GCATGTCTGGCTTGCACCTAG
	Reverse	CATCTTAATGTATTCTGCATTGGC

Gene full names. RPLPO: Ribosomal protein P0; LXR: Liver X receptor; CD36: Cluster of differentiation 36; PPARα: Peroxisome proliferator.activated receptor; SREBP: sterol regulatory element-binding protein; FASN: Fatty acid synthase; IL-1β: Interleukin 1 beta; IL-6: Interleukin 6; CPT-1: Carnitine palmitoyltransferase; ACC: Acetyl-CoA-carboxylase.
